# Enhancing variant detection in complex genomes: leveraging linked reads for robust SNP, Indel, and structural variant analysis

**DOI:** 10.21203/rs.3.rs-8408441/v1

**Published:** 2026-01-12

**Authors:** Can Luo, Yichen Henry Liu, Han Liu, Zhenmiao Zhang, Lu Zhang, Brock A. Peters, Xin Maizie Zhou

**Affiliations:** 1 Department of Biomedical Engineering, Vanderbilt University, 37235 Nashville, USA.; 2 Department of Computer Science, Vanderbilt University, 37235 Nashville, USA.; 3 Department of Computer Science and Engineering, University of California San Diego, 92037 San Diego, USA.; 4 Department of Computer Science, Hong Kong Baptist University, Hong Kong, China.; 5 Advanced Genomics Technology Lab, Complete Genomics Inc, 2904 Orchard Parkway, San Jose, CA 95134, USA.

**Keywords:** Linked-read sequencing, Sequencing simulation, SNP call, INDEL call, Structural variants call, stLFR

## Abstract

**Background:**

Accurate detection of genetic variants, including single nucleotide polymorphisms (SNPs), small insertions and deletions (INDELs), and structural variants (SVs), is critical for comprehensive genomic analysis. While traditional short-read sequencing performs well for SNP and INDEL detection, it struggles to resolve SVs, especially in complex genomic regions, due to inherent read length limitations. Linked-read sequencing technologies, such as single-tube Long Fragment Read sequencing (stLFR), overcome these challenges by employing molecular barcodes, providing crucial long-range information.

**Methods:**

This study investigates traditional pair-end linked-reads and a conceptual extension of linked-read technology: barcoded single-end reads of 500 bp (SE500_stLFR) and 1000 bp (SE1000_stLFR), generated using the single-tube Long Fragment Read (stLFR) platform. Unlike conventional paired-end (PE100_stLFR) linked reads, these longer single-end reads could offer improved resolution for variant detection by leveraging extended read lengths per barcode. To explore the potential of stLFR reads, we developed stLFR-sim, a Python-based simulator that reproduces the stLFR linked-read sequencing workflow to enable realistic simulation and benchmarking of linked-read sequencing data. Using stLFR-sim, we simulated a diverse set of datasets for the HG002 sample using T2T-based realistic genome simulation. Variant detection performance was then systematically assessed across three stLFR configurations: standard PE100_stLFR, SE500_stLFR, and SE1000_stLFR.

**Results:**

Benchmarking against the Genome in a Bottle (GIAB) gold standard reveals distinct strengths of each configuration. Extended single-end reads (SE500_stLFR and SE1000_stLFR) significantly enhance SV detection, with SE1000_stLFR providing the best balance between precision and recall. In contrast, the shorter PE100_stLFR reads exhibit higher precision for SNP and INDEL calling, particularly within high-confidence regions, though with reduced performance in low-mappability contexts. To explore optimization strategies, we constructed hybrid libraries combining paired-end and single-end barcoded reads. These hybrid approaches integrate the complementary advantages of different read types, consistently outperforming single libraries across small variant types and genomic contexts.

**Conclusion:**

Collectively, our findings offer a robust comparative framework for evaluating stLFR sequencing strategies, highlight the promise of barcoded single-end reads for improving SV detection, and provide practical guidance for tailoring sequencing designs to the complexities of the genome.

## Background

Advancements in sequencing technologies have revolutionized genomics study by enabling precise and high-throughput analysis of DNA, offering unprecedented opportunities to understand genetic variation [1], disease mechanisms [2], and evolutionary biology [3]. Among these advancements, short-read sequencing [4,5] has become the backbone of many genomic studies due to its cost-effectiveness, scalability, and high accuracy in detecting small variants, such as single nucleotide polymorphisms (SNPs) and small insertions and deletions (indels). However, despite its transformative impact, the intrinsic limitations of short-read sequencing in terms of read length impose significant challenges when addressing more complex genomic features, such as structural variants (SVs), repetitive regions, and chromosomal rearrangements. The inability to span long repetitive sequences or phase variants across distant loci restricts its utility for comprehensive genome characterization, leaving gaps in our understanding of genetic variation.

To overcome these challenges, innovative technologies have been developed to extend the capabilities of sequencing, providing long-range information and improved resolution of complex genomic features. One notable advancement is linked-read sequencing, which combines short-read sequencing with molecular barcoding to retain long-range context [6,7]. This method starts by fragmenting high molecular weight (HMW) DNA into long segments, typically tens to hundreds of kilobases in length. Each fragment is encapsulated into microfluidic droplets or physically separated into individual compartments, where it undergoes amplification and tagging with a unique molecular barcode. Importantly, all short reads derived from a single DNA fragment carry the same unique barcode, allowing them to be computationally grouped and assigned to their original long DNA molecule. Once the barcoded DNA fragments are sequenced, bioinformatics tools can reconstruct the long-range structure of the original DNA by clustering reads based on their shared barcode [8-10]. This enables linked-read sequencing to effectively span long regions of the genome, even with the relatively short read lengths produced by sequencing platforms like Illumina. This methodology enhances genome assembly, variant detection, and haplotype phasing by preserving the positional information of DNA fragments, which is critical for resolving structural variants and complex regions [11-15].

Among linked-read technologies, the 10x Genomics Chromium platform stands out as a pioneering implementation [6]. It uses microfluidics to partition HMW DNA into thousands of droplets, each containing a unique barcode and reagents for DNA amplification. This approach ensures high efficiency and scalability, enabling the analysis of large and complex genomes. However, one limitation of this method is the occurrence of multiple DNA molecules within a single droplet, leading to potential barcode collisions and reduced resolution in some cases. The advent of single-tube long fragment read (stLFR) technology addressed this issue by eliminating the need for droplet-based separation and ensuring a “near” one-to-one correspondence between individual DNA molecules and barcodes [7]. Using a bead-based chemistry, stLFR introduces barcodes directly to DNA fragments in solution, simplifying the workflow and improving the accuracy of molecule-to-barcode association. This innovation further enhances the resolution of linked-read sequencing, particularly for applications requiring high precision in structural variant detection and haplotype phasing. Despite these advancements, there remains a need to explore novel strategies that can further optimize long-range genomic insights while addressing challenges in library preparation, sequencing efficiency, and resolution.

Building on these developments, this study investigates a conceptual extension of linked-read technology: barcoded single-end reads of 500 bp and 1000 bp. Unlike traditional paired-end reads commonly used in linked-read sequencing, barcoded single-end reads have the potential to simplify library preparation workflows while leveraging longer individual reads per barcode to provide enhanced resolution. This approach holds promise for improving SV detection, particularly in genomic regions that are challenging for short-read and paired-end technologies. By systematically evaluating the impact of sequencing parameters on variant detection using stLFR-sim simulated paired-end and conceptual single-end barcoded reads, this study leverages the stLFR framework as a comparative benchmark. The findings aim to inform future developments in sequencing technology and provide valuable insights into optimizing genomic analyses for complex regions and structural variations.

## Methods

### Simulator pipeline

In this study, we present stLFR-sim, a Python-based simulator designed to generate linked-read sequencing data that mimics the output of the stLFR platform and Illumina sequencers. While it draws partial inspiration from the methodology of LRTK-sim [16], stLFR-sim is an original tool that introduces several novel features, described in detail in the subsections below. The simulator reproduces the stLFR linked-read sequencing workflow through four main steps: (1) generating a diploid reference genome, (2) simulating long DNA fragments, (3) assigning barcodes to DNA fragments, and (4) generating barcoded Illumina short reads. This pipeline enables realistic simulation of linked-read data, making stLFR-sim a valuable resource for benchmarking sequencing workflows and downstream analysis tools. Each step is elaborated below to provide a comprehensive overview of the simulation process.

#### 1. Generating the Diploid Reference Genome

stLFR-sim begins with a sample-specific diploid genome as input. While the original LRTK-sim pipeline generated this by inserting phased, high-confidence variants into a reference genome, our approach instead utilizes a phased, realistic diploid assembly from the HG002 sample (see section titled “T2T Realistic Simulation” for details). This diploid reference serves as the foundation for generating synthetic linked-read data, allowing the simulation to capture the genomic complexity of a real individual.

#### 2. Simulating Long DNA Fragments

With the diploid genome defined, stLFR-sim proceeds to simulate long DNA fragments. The physical coverage, denoted $C_{F}$ , represents the cumulative coverage of all DNA fragments across the genome. The total amount of DNA, V, is calculated as $V=C_{F}\times L$, where L is the length of the reference genome. To model both haplotypes equally, each is assigned half of the total physical coverage, i.e., $C_{F}∕2$. Fragment start positions are randomly distributed across the genome, and fragment lengths are drawn from an exponential distribution with a mean of $\mu_{FL}$. This step ensures that the simulated fragment size distribution closely mirrors that observed in real linked-read sequencing data.

#### 3. Barcoding DNA Fragments

In this step, stLFR-sim emulates the barcoding process used in stLFR technology. There are originally 1536 unique 10-mer barcodes in the stLFR barcode list. stLFR-sim randomly selects 3 barcodes from the list, thereby generating up to 3.62 billion unique 30-mer barcode sequences for labeling DNA fragments. This number (3.62 billion) represents the theoretical maximum number of barcodes; however, the actual number utilized depends on the values of $C_{F}$ and $\mu_{FL}$, and is typically much lower than the maximum. Specifically, the actual number of barcodes can be estimated as $N_{bc}=C_{F}\times \text{genome size}\;∕\;\mu_{FL}$. stLFR-sim generates “pseudo” partitions, each assigned a unique barcode.

In contrast to LRTK-sim’s pipeline, which simulates the stochastic barcoding process by assigning multiple fragments to a single partition, stLFR-sim reflects the design of stLFR technology, which enables precise assignment of one fragment per partition. Consequently, each DNA fragment in stLFR-sim is assigned to a distinct partition with a unique barcode, accurately modeling the one-fragment-per-barcode characteristic of stLFR.

#### 4. Simulating Barcoded Illumina Short Reads

The final step in stLFR-sim involves generating barcoded Illumina short reads to cover the simulated long DNA fragments. The total number of reads is calculated as $\frac{C_{R}\times V}{R_{L}}$, where $C_{R}$ denotes the desired read coverage, V is the total DNA volume, and $R_{L}$ is the read length, typically set to 150 bp by default. Reads are uniformly distributed across each fragment to mimic even sequencing coverage.

Paired-end short reads are generated with insert sizes sampled from a normal distribution centered at a user-defined mean $\mu_{IS}$. Short reads are tagged with barcodes derived from their associated DNA fragments. To further enhance realism, stLFR-sim incorporates empirical base quality profiles derived from linked-read datasets sequenced on an Illumina HiSeq X platform. Sequencing errors are introduced at random positions based on these quality scores, providing a more accurate simulation of real-world sequencing data.

#### 5. Simulating Other Sequencing Data Types

To broaden the scope of the simulation, stLFR-sim also supports the generation of standard Illumina short reads (without barcodes) and single-end barcoded reads. For simulating unbarcoded Illumina short reads, the barcoding step is simply disabled while the rest of the pipeline remains unchanged. To simulate single-end barcoded reads, the paired-end sequencing module is replaced with a single-end mode, which randomly samples fixed-length reads from each fragment.

In summary, stLFR-sim provides several advantages over existing linked-read simulators. First, it allows users to flexibly configure key parameters, including $C_{F}$, $C_{R}$, $\mu_{FL}$, $R_{L}$, and $\mu_{IS}$, to accurately model specific experimental conditions. Second, we have extended its functionality to support the simulation of standard Illumina short reads (without barcodes) and single-end barcoded reads, enhancing its versatility. Finally, stLFR-sim is a lightweight, all-in-one Python package that requires no third-party software. In contrast to simulators like LRSIM, which depend on multiple external tools, stLFR-sim is self-contained and capable of simulating multiple libraries in a single run, making it both efficient and user-friendly for exploring a wide range of experimental designs.

### T2T realistic simulation

To simulate reads sequenced within realistic genomic contexts, we utilized the phased diploid assembly of HG002 released by the Human Pangenome Reference Consortium (HPRC) [17]. The paternal and maternal haplotype assemblies were independently aligned to the T2T-CHM13v2.0 reference using minimap2. Contigs from each haplotype that aligned to the target chromosome (e.g., chromosome 6) were then extracted using SAMtools and used as input for our simulation.


minimap2 --secondary=no -a --eqx -Y -
   x asm20 -s 200000 -z 10000 ,50 -r
   50000 --end - bonus =100 -O 5,56 -E
   4 , 1 -B 5 ${ref} ${asm} ∣ samtools
   sort -o ${bam}


### Simulation configurations

Read libraries were simulated under various configurations to systematically investigate the impact of different parameter combinations. Libraries labeled as 35x or 70x correspond to read coverages of 35x and 70x, respectively. Both PE100_NGS and PE100_stLFR libraries consist of paired-end reads with a length of 100 bp, while SE500_stLFR and SE1000_stLFR libraries contain single-end reads with lengths of 500 bp and 1000 bp, respectively. A total of 12 simulation experiment (Exp1-Exp12) were conducted for each library type. These experiments were categorized into three groups: Exp1-Exp4, Exp5-Exp8, and Exp9-Exp12. Within each group, the parameters $C_{F}$ and $C_{R}$ were varied, while the parameter $\mu_{FL}$ differed between groups. All groups shared consistent values for insertion size, error rate, and $N_{FL}$.

More specifically, for 35x coverage experiments, the $C_{F}\text{-}C_{R}$ combinations ranged from 350-0.1 to 47-0.75 within each group. For the 70x coverage experiments, the $C_{F}$ values were doubled. The $\mu_{FL}$ values were set to 50kb, 75kb, and 100kb for Exp1-Exp4, Exp5-Exp8, and Exp9-Exp12, respectively. Across all experiments, the insertion size, error rate, and $N_{FL}$ were consistently set to 600 bp, 1%, and 1, respectively. Full simulation configurations are summarized in Table ??.

### SV calling

We developed Aquila_stLFR v1.1 as an updated pipeline for structural variant (SV) calling, based on the original Aquila_stLFR framework [9]. This version is a reference-assisted tool designed to perform local *de novo* assembly of stLFR linked-read data. It reconstructs haplotype-specific blocks using barcode-tagged long fragment reads (LFRs) and performs localized assembly within small genomic regions to achieve high contiguity and accurate SV detection.

Aquila stLFR v1.1 processes stLFR data in three main steps: (1) Haplotype phasing: Long fragment reads (LFRs) are reconstructed and partitioned into haplotype-specific blocks using barcode information and heterozygous SNPs. (2) Local assembly: Within 100 kb genomic chunks, reads are locally assembled using SPAdes. Resulting mini-contigs are then iteratively merged to form full-length haplotype-resolved contigs. (3) SV detection: Structural variants are identified by aligning haploid contigs to the reference genome using Minimap2 [18], followed by a custom SV detection pipeline based on VolcanoSV-vc [19].

While steps 1 and 2 remain unchanged from the original Aquila stLFR, step 3 has been enhanced in version 1.1 to incorporate the VolcanoSV-vc methodology for more robust SV detection.

VolcanoSV-vc is the variant calling module of VolcanoSV [19], originally designed for long-read sequencing data. For our linked-read application, we adapted VolcanoSV-vc by disabling the long-read-specific signature scanning module. In our workflow, VolcanoSV-vc detects SVs from haplotype-resolved contigs aligned to the reference genome using Minimap2. SVs, such as large insertions and deletions, are identified based on CIGAR string information and split alignment signatures.

SV signatures are collected independently for each haplotype, clustered using a nearest-neighbor algorithm, and refined to eliminate redundancy. Within each cluster, the longest signature is retained as the final SV call. Genotypes are then assigned based on the presence of SVs across haplotypes: “1∣1” if present in both, and “0∣1” or “1∣0” if present in only one. Despite potential assembly artifacts, VolcanoSV-vc uses empirically optimized thresholds to ensure accurate clustering and genotyping in the linked-read context.

The following commands were used to generate the SV calls.


Aquila_stLFR1 .1 _step1 .py \
    --fastq_file ${ reads_fastq } \
    --bam_file ${ bamfile } \
    --vcf_file ${ SNP_vcf } \
    --sample_name NA24385 \
    --out_dir ${ outdir } \
    --uniq_map_dir ./Aquila_stLFR/ Uniqness_map_hg19 \
       Uniqness_map_hg19 \
    --chr_start ${ chr_num } -- chr_end
        ${ chr_num } --num_threads $t -r $ dtype
       r $ dtype
Aquila_stLFR1 .1 _step2 .py \
    --out_dir ${ outdir } \
    --num_threads $t \
    --reference $ reference \
    --chr_start ${ chr_num } -- chr_end
       ${ chr_num } -r $ dtype
Aquila_stLFR1 .1 _step3 .py \
        -contig ${ outdir }/
           Assembly_Contigs_files /
	   Aquila_Contig_chr${ chr_num }. fasta \
	   }. fasta \
	-ref ${ reference } \
        -o ${ outdir }/ SV_ouput / \
	-chr ${ chr_num } -t $t


In addition, Aquila stLFR v1.1 introduces support for processing barcoded single-end reads. This functionality is enabled by modifying the read processing module in Step 1, where the basic unit for haplotype estimation is changed from read pairs to individual single-end reads. Users can specify the data type using the ‘-r’ flag, with ‘PE’ for paired-end and ‘SE’ for single-end reads.

### SNP and small INDEL calling

For single-library SNP and INDEL calling, simulated reads were first aligned to the reference genome using BWA-MEM for NGS or stLFR single-end reads, and EMA for stLFR paired-end reads. In the case of hybrid simulations, final alignments were generated by merging BAM files from the two individual libraries. Read group information was then added to the alignments involving NGS reads using Picard. Finally, SNP and INDEL calling was performed using the GATK pipeline.

The following commands were used to generate the SNP and INDEL calls:


bwa mem \
    ${ reference } \
    ${ R1_fastq } \
    ${ R2_fastq } \
    -t 30 ∣ samtools sort -o ${ bwa_bam }
       }
ema al ign \
    -1 ${ R1_fastq } \
    -2 ${ R2_fastq } \
    -r  ${ reference } -t 30 ∣ samtools
        sort -@ 30 -o ${ ema_bam }
java -jar picard .jar
   AddOrReplaceReadGroups \
    I=${ raw_bamfile } \
    O=${ bamfile } \
    RGID =4 \
    RGLB = lib1 \
    RGPL = ILLUMINA \
    RGPU = unit1 \
    RGSM = 20
gatk HaplotypeCaller -I $ bamfile -O
   gatk .vcf -R ${ reference }


### Benchmarking

For SV detection evaluation, we used Truvari [20] to benchmark our SV calls against the GIAB HG002 SV Tier1 v0.6 high-confidence truth set on the hg19 reference genome. Truvari was configured with the following parameters: maximum reference location distance of 500 bp (*refdist*), minimum allele sequence similarity of 50% (*pctsim*), minimum allele size similarity of 50% (*pctsize*), minimum reciprocal overlap of 1% (*pctovl*), and restriction to high-confidence regions (*includebed*) and SVs labeled as PASS (*passonly*). All other parameters were left at default values.

SNP and INDEL benchmarking was performed using hap.py against the NIST v4.2.1 HG002 GRCh37 call set on the hg19 reference. Analyses in both high-confidence and difficult genomic regions, including the MHC, tandem and homopolymer repeats (tandemRep), segmental duplications (segdup), and low-mappability regions (lowmap), were conducted using the corresponding BED files from the NIST v4.2.1 genome stratifications. Default parameters were used for all hap.py evaluations.

## Results

To evaluate the performance of linked-read sequencing under controlled conditions, we developed stLFR-sim, a Python-based simulator that replicates the stLFR sequencing process. The tool generates diploid reference genomes, simulates DNA fragmentation and barcode assignment, and produces barcoded short reads that closely resemble real stLFR data. stLFR-sim also offers the option to turn off barcode assignment and generate regular Illumina short reads. This realistic and versatile simulation framework allows flexible simulation configuration and thus facilitates comprehensive downstream benchmark study.

Leveraging stLFR-sim, we mainly simulated three kinds of sequencing data: traditional Illumina short paired-end reads, stLFR paired-end reads and stLFR single-end reads. To comprehensively assess the boundaries and potential of single-end stLFR reads with extended read lengths of 500 bp (SE500_stLFR) and 1000 bp (SE1000_stLFR), we compared them against traditional 100 bp paired-end stLFR reads (PE100_stLFR). We first evaluated the SNP and INDEL calling performance using libraries generated from each individual stLFR sequencing technology (single libraries). We then explored the potential benefits of hybrid libraries combining paired-end next-generation sequencing (NGS) reads, paired-end stLFR reads, and single-end stLFR reads. This approach tested our hypothesis that hybrid libraries could outperform single libraries in SNP and INDEL detection within complex genomic regions by leveraging the complementary strengths of longer single-end reads and paired-end reads. Additionally, we assessed structural variant (SV) calling performance in single libraries, emphasizing the enhanced SV detection capability of SE500_stLFR and SE1000_stLFR.

To enable comprehensive comparisons, we generated 12 simulation experiments (Exp1-Exp12) for each library type under various settings and sequencing depths, totaling 84 simulations. These experiments were grouped as Exp1-Exp4, Exp5-Exp8, and Exp9-Exp12. Within each group, the physical coverage $C_{F}$ and the desired read coverage $C_{R}$ were varied, while the mean fragment length $\mu_{FL}$ differed between groups. All experiments used the same insertion size (600 bp), error rate (1%), and the average number of molecules per droplet $N_{FL}$ (see Table ?? and the Simulation Configurations section).

Given the scale of the simulation data, we limited our analysis to chromosome 6 to allow a more detailed, fine-grained evaluation, where we stratified the genomic regions to high-confidence regions (allconf) and challenging (difficult) regions (alldiff). The alldiff regions were further subdivided into four categories representing specific genomic complexities: low-mappability (lowmap), segmental duplications (segdup), tandem and homopolymer repeats (tandemRep), and major histocompatibility complex (MHC) regions.

### SNP and INDEL calling performance across different single linked-read library types

**Figure 1:**
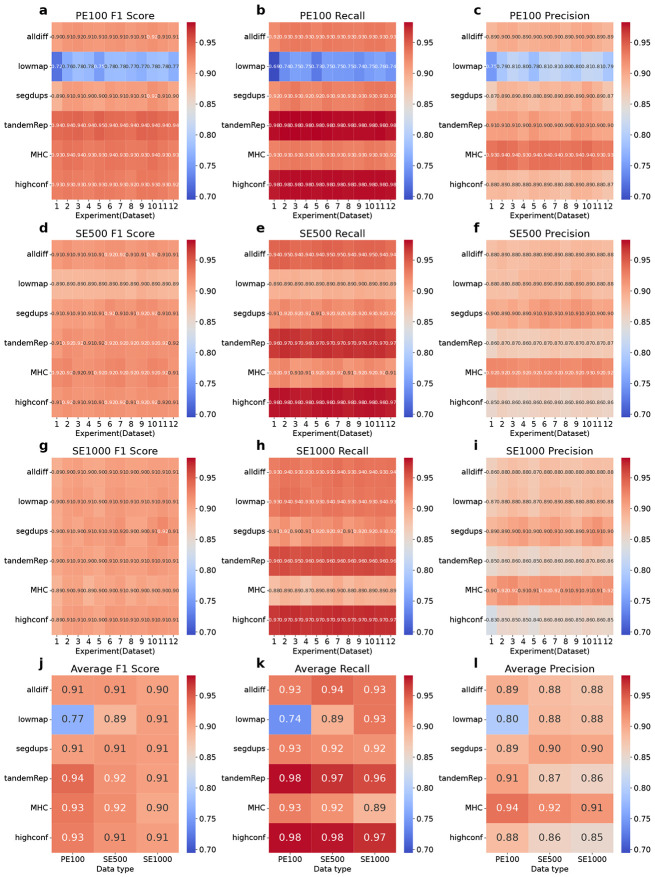
Single-library (35x) SNP calling performance across different genomic regions. The SNP calls of three single libraries (35x) was evaluated in terms of F1 Score, Recall, and Precision across a range of genomic contexts, including all difficult (alldiff), low-mappability (lowmap), segmental duplication (segdup), tandem and homopolymer repeat (tandemRep), MHC, and high-confidence (highconf) regions. **a-c.** SNP evaluation results for the PE100_stLFR 35x library. **d-f.** SNP evaluation results for the SE500_stLFR 35x library. **g-i.** SNP evaluation results for the SE1000_stLFR 35x library. For each single library, 12 experiments (datasets) were simulated to explore the impact of sequencing parameters on variant detection. **j-l.** Average SNP call F1 score, recall and precision for each data type across different genomic regions.

**Figure 2:**
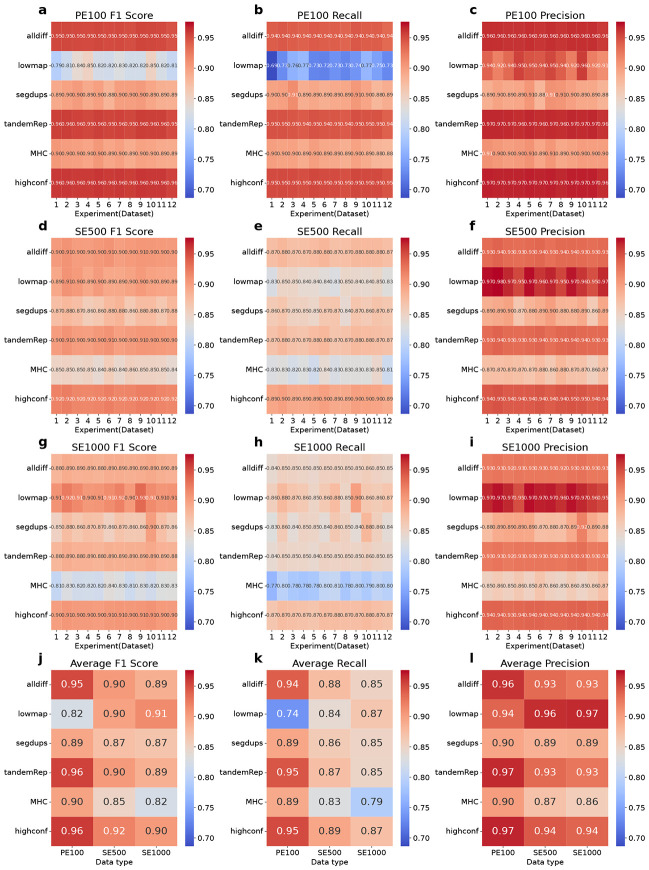
Single-library (35x) INDEL calling performance across different genomic regions. The SNP calls of three single libraries (35x) was evaluated in terms of F1 Score, Recall, and Precision across a range of genomic contexts, including all difficult (alldiff), low-mappability (lowmap), segmental duplication (segdup), tandem and homopolymer repeat (tandemRep), MHC, and high-confidence (highconf) regions. **a-c.** INDEL evaluation results for the PE100_stLFR 35x library. **d-f.** INDEL evaluation results for the SE500_stLFR 35x library. **g-i.** INDEL evaluation results for the SE1000_stLFR 35x library. For each single library, 12 experiments (datasets) were simulated to explore the impact of sequencing parameters on variant detection. **j-l.** Average INDEL call F1 score, recall and precision for each data type across different genomic regions.

In this section, we comprehensively evaluated SNP and INDEL calling results across three types of single stLFR linked-read libraries (PE100_stLFR, SE500_stLFR, and SE1000_stLFR) within four challenging (difficult) genomic regions: low-mappability (lowmap), segmental duplications (segdup), tandem and homopolymer repeats (tandemRep), and major histocompatibility complex (MHC), as well as all difficult regions combined (alldiff) and high-confidence regions (allconf). This analysis elucidates each type of single library’s ability to capture small genetic variations under diverse genomic contexts (Figure 1-2). For each single library type, we examined 12 datasets to explore the impact of sequencing parameters on small variant calling.

Overall, PE100_stLFR demonstrates superior performance under most conditions. However, the longer read lengths of SE500_stLFR and SE1000_stLFR offer distinct advantages, particularly for SNP and INDEL detection in low-mappability (lowmap) regions and SNP detection in segmental duplication (segdup) regions.

In SNP analysis, the general trend in high-confidence regions indicates that PE100_stLFR outperforms SE500_stLFR and SE1000_stLFR. Specifically, PE100_stLFR demonstrates the highest F1 score, averaging 0.93, while SE500_stLFR and SE1000_stLFR yield scores around 0.91 (Figure 1a, d, and g). This difference primarily arises from variations in precision, as the recall rates remain consistent across all three types of libraries, ranging from 0.97 to 0.98. PE100_stLFR achieves an average precision of 0.88, notably higher than SE500_stLFR (0.86) and SE1000_stLFR (0.85) (Figure 1c, f, and i).

Across all difficult regions, SNP detection performance is generally comparable among the three library types. The average F1 scores are 0.91 for PE100_stLFR and SE500_stLFR, and 0.90 for SE1000_stLFR (Figure 1j). However, performance varies across specific challenging regions. In tandem and homopolymer repeats (tandemRep) regions, PE100_stLFR demonstrates superior performance, achieving an average F1 score of 0.94, compared to 0.92 for SE500_stLFR and 0.91 for SE1000_stLFR (Figure 1j). This advantage is largely due to PE100_stLFR’s higher average precision of 0.91, outperforming SE500_stLFR (0.87) and SE1000_stLFR (0.86) (Figure 1l). Similarly, in MHC regions, PE100_stLFR leads with an F1 score of 0.93, followed by SE500_stLFR (0.92) and SE1000_stLFR (0.90) (Figure 1j). Conversely, in low-mappability (lowmap) regions, SE500_stLFR and SE1000_stLFR exhibit better performance than PE100_stLFR, with recall rates improving as read lengths increase (Figure 1j-k). SE1000_stLFR achieves the highest F1 score (0.91), followed by SE500_stLFR (0.89), while PE100_stLFR lags significantly with an F1 score of 0.77 (Figure 1j). In segmental duplication (segdup) regions, SE500_stLFR (F1: 0.91) and SE1000_stLFR (F1: 0.908) exhibit a slight advantage over PE100_stLFR (F1: 0.906), primarily due to PE100_stLFR’s lower precision (Figure 1j-l).

In INDEL analysis, PE100_stLFR consistently demonstrates superior overall performance across both high-confidence and all difficult regions, as indicated by higher F1 scores compared to SE500_stLFR and SE1000_stLFR (Figure 2a, d, and g). In high-confidence regions, PE100_stLFR achieves an average F1 score of 0.96, significantly outperforming SE500_stLFR (0.92) and SE1000_stLFR (0.90) (Figure 2j). A similar trend is observed across all difficult regions, where PE100_stLFR attains an average F1 score of 0.95, compared to 0.90 for SE500_stLFR and 0.89 for SE1000_stLFR (Figure 2j). This disparity in F1 scores is largely due to the notably lower recall rates observed in SE500_stLFR and SE1000_stLFR (Figure 2b, e, h and k). In high-confidence regions, SE500_stLFR and SE1000_stLFR yield average recall rates of 0.89 and 0.87, respectively, whereas in all difficult regions, their recall rates drop further to 0.88 and 0.85. In contrast, PE100_stLFR maintains higher recall rates, averaging 0.95 in high-confidence regions and 0.94 in difficult regions (Figure 2k).

Further analysis of specific difficult regions reveals that PE100_stLFR outperforms SE500_stLFR and SE1000_stLFR in segmental duplication (segdup), tandem and homopolymer repeats (tandemRep), and MHC regions, with recall rate differences driving this trend. In segmental duplication (segdup) regions, PE100_stLFR achieves an average F1 of 0.89, compared to 0.87 for both SE500_stLFR and SE1000_stLFR (Figure 2j). In tandem and homopolymer repeats (tandemRep) regions, PE100_stLFR achieves an average F1 of 0.95, outperforming SE500_stLFR (0.90) and SE1000_stLFR (0.89). Similarly, in MHC regions, PE100_stLFR achieves an F1 score of 0.90, outperforming SE500_stLFR (0.85) and SE1000_stLFR (0.82). Conversely, SE500_stLFR and SE1000_stLFR demonstrate superior performance in low-mappability (lowmap) regions, where recall rates improve with increasing read length. SE500_stLFR and SE1000_stLFR achieve average F1 scores of 0.90 and 0.91, respectively, both exceeding PE100_stLFR’s F1 score of 0.82. This advantage primarily stems from their higher recall rates, with SE500_stLFR and SE1000_stLFR averaging 0.84 and 0.87, respectively, compared to 0.73 for PE100_stLFR (Figure 2k). Furthermore, both SE500_stLFR and SE1000_stLFR maintain higher precision than PE100_stLFR in low-mappability (lowmap) regions, with average precision scores of 0.96 and 0.97, respectively, while PE100_stLFR averages 0.94 (Figure 2l).

As previously mentioned, 12 datasets (experiments) with varying sequencing parameters were used to evaluate variant calling for each single library type. Specifically, the impact of different combinations of $C_{F}$ and $C_{R}$ on SNP and INDEL calling was assessed. The parameter $C_{F}$ ranged from 350 to 47, while $C_{R}$ varied from 0.1 to 0.75, with the total coverage consistently maintained at 35x. Overall, the results show a slight performance improvement with intermediate configurations ($C_{F}\text{-}C_{R}$ values of 140-0.25 or 70-0.5); however, no clear trend emerges (Figure ??).

### SNP and INDEL calling performance across different hybrid linked-read library types

Longer single-end reads are advantageous for detecting structural variants but tend to exhibit lower accuracy in identifying SNPs and INDELs. In contrast, shorter paired-end reads excel in accurately detecting small variants but face challenges in capturing large structural variants due to their limited length. Given these trade-offs, it is valuable to explore whether combining different data types can effectively leverage the strengths of both data types. In this session, we designed a series of hybrid library experiments to compare them with single-library experiments. For hybrid library experiments, we created three types of hybrid libraries: (1) PE100_NGS (35x) combined with PE100_stLFR (35x), (2) SE500_stLFR (35x) combined with PE100_stLFR (35x), and (3) SE1000_stLFR (35x) combined with PE100_stLFR (35x). For each type of hybrid library, we used 12 datasets to explore the impact of sequencing parameters. For single-library experiments, we also used 12 corresponding datasets for PE100_NGS, PE100_stLFR, SE500_stLFR, and SE1000_stLFR, each with a total coverage of 70x, and evaluated their respective performance.

Overall, in both SNP and INDEL analyses, hybrid libraries demonstrate strong performance, often outperforming or ranking between their corresponding single libraries. However, their effectiveness varies by region and depends on whether precision or recall is the limiting factor. Further details are discussed below.

**Figure 3:**
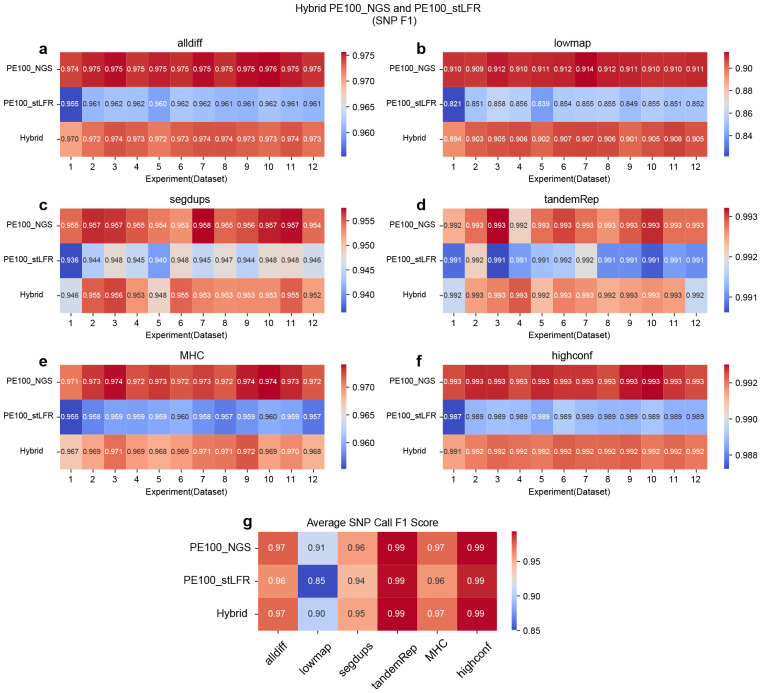
F1 accuracy heatmap for SNP calling on hybrid libraries by combing PE100_NGS and PE100_stLFR reads. *hap.py* v0.3.15 was applied to benchmark the SNP callset against the GIAB ground truth callset. **a.** F1 accuracy for all difficult (alldiff) regions. **b.** F1 accuracy for low-mappability (lowmap) regions. **c.** F1 accuracy for segmentation duplication (segdup) regions. **d.** F1 accuracy for tandem and homopolymer repeat (tandemRep) regions. **e.** F1 accuracy for MHC regions. **f.** F1 accuracy for all high-confidence (highconf) regions defined by GIAB. **g.** Average SNP call F1 score for each library across different genomic regions.

**Figure 4:**
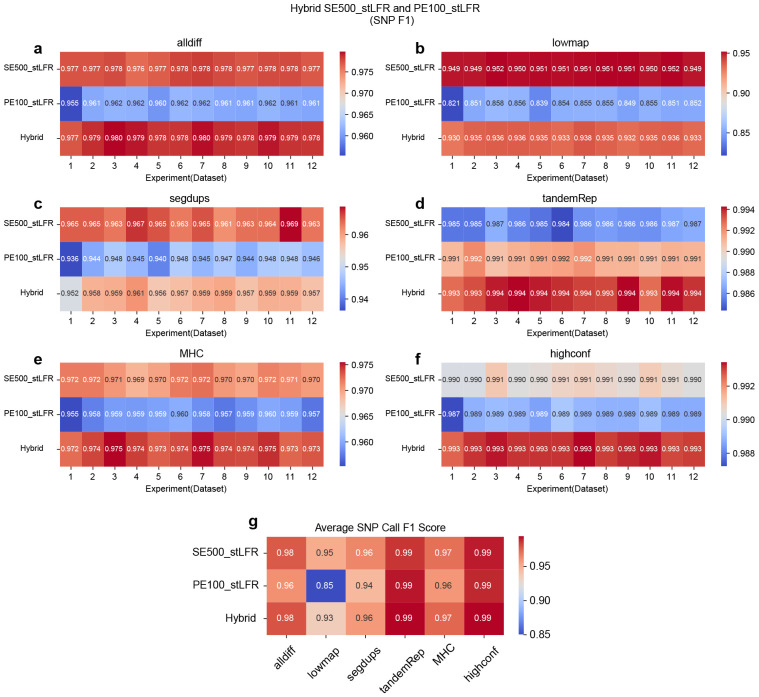
F1 accuracy heatmap for SNP calling on hybrid libraries by combing SE500_stLFR and PE100_stLFR reads. *hap.py* v0.3.15 was applied to benchmark the SNP callset against the GIAB ground truth callset. **a.** F1 accuracy for all difficult (alldiff) regions. **b.** F1 accuracy for low-mappability (lowmap) regions. **c.** F1 accuracy for segmentation duplication (segdup) regions. **d.** F1 accuracy for tandem and homopolymer repeat (tandemRep) regions. **e.** F1 accuracy for MHC regions. **f.** F1 accuracy for all high-confidence (highconf) regions defined by GIAB. **g.** Average SNP call F1 score for each library across different genomic regions.

**Figure 5:**
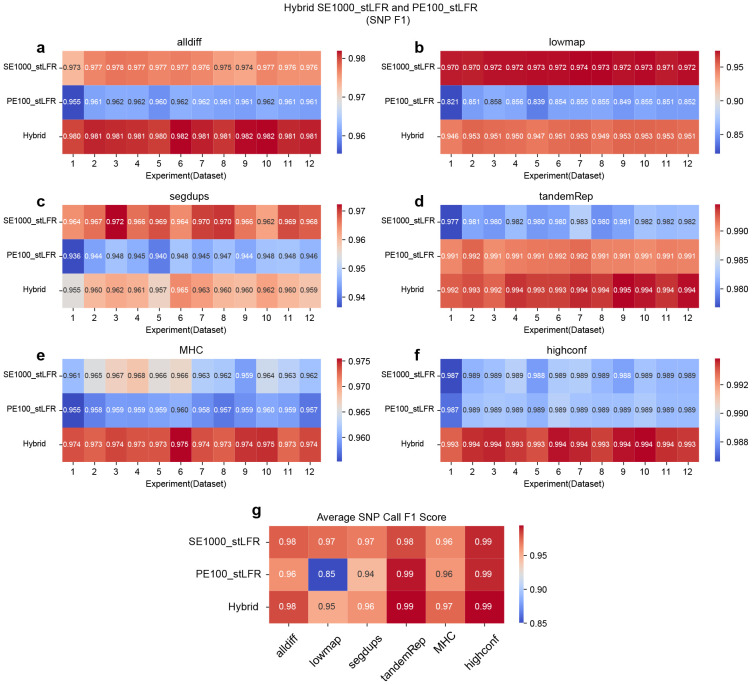
F1 accuracy heatmap for SNP calling on hybrid libraries by combing SE1000_stLFR and PE100_stLFR reads. *hap.py* v0.3.15 was applied to benchmark the SNP callset against the GIAB ground truth callset. **a.** F1 accuracy for all difficult (alldiff) regions. **b.** F1 accuracy for low-mappability (lowmap) regions. **c.** F1 accuracy for segmentation duplication (segdup) regions. **d.** F1 accuracy for tandem and homopolymer repeat (tandemRep) regions. **e.** F1 accuracy for MHC regions. **f.** F1 accuracy for all high-confidence (highconf) regions defined by GIAB. **g.** Average SNP call F1 score for each library across different genomic regions.

In SNP calling, hybrid libraries exhibit varying performance depending on the combination of single library types and genomic regions. For the combination of PE100_NGS and PE100_stLFR, hybrid libraries generally show intermediate performance across all regions. Precision is reduced in low-mappability (lowmap), segmental duplication (segdup), and tandem and homopolymer repeat (tandemRep) regions, while both recall and precision decline in all difficult (alldiff), MHC, and high-confidence (highconf) regions (Figure 3 and Figure ??-??). For the combination of SE500_stLFR and PE100_stLFR, hybrid libraries perform better than both corresponding single libraries in four regions (alldiff, tandemRep, MHC, and highconf). However, performance is intermediate in lowmap and segdups regions due to reductions in both precision and recall (Figure 4 and Figure ??-??). Similarly, for the SE1000_stLFR and PE100_stLFR combination, hybrid libraries outperform both corresponding single libraries in alldiff, tandemRep, MHC, and highconf, but demonstrate intermediate performance in lowmap and segdups, where both recall and precision are affected, particularly in lowmap (Figure 5 and Figure ??-??). Across all experiments, hybrid libraries perform particularly well in all highconf regions and challenging regions like tandemRep, suggesting that combining barcoded short and long reads can enhance SNP calling.

For INDEL detection, the results follow a similar trend. In the PE100_NGS and PE100_stLFR combination, hybrid libraries generally outperform or match their corresponding single libraries across most regions (Figure ??-??). They achieve superior performance in regions such as alldiff, lowmap, tandemRep, and highconf but rank between the two corresponding single libraries in MHC due to lower recall and in segdups due to lower precision. For the SE500_stLFR and PE100_stLFR combination, hybrid libraries show the best performance in alldiff, segdups, MHC, and highconf (Figure ??-??). However, their performance in lowmap and tandemRep is intermediate, primarily due to reduced precision. A similar pattern was observed for the SE1000_stLFR and PE100_stLFR combination, where hybrid libraries perform best in segdups and MHC. However, they show reduced precision in alldiff and highconf, along with decreases in both recall and precision in lowmap and tandemRep (Figure ??-??).

Overall, these findings suggest that hybrid libraries can offer advantages over single libraries in certain instances, especially in regions where short or long reads alone struggle to perform optimally. By combining short paired-end reads with longer barcoded single-end reads, hybrid libraries help mitigate the limitations of each approach, enhancing the detection of both SNPs and INDELs. However, the intermediate performance observed in some regions reflects the trade-off between precision and recall, emphasizing the importance of balancing these factors when designing hybrid libraries for different genomic contexts.

### SV calling performance across different single linked-read library types

In this session, we evaluated the performance of structural variant (SV) calling across three types of single linked-read libraries: PE100_stLFR, SE500_stLFR, and SE1000_stLFR. Each sequenced at 35x coverage. For each library type, we analyzed 12 datasets to investigate the effects of sequencing parameters on SV calling. SVs were identified using by Aquila_stLFR, a tool tailored for linked-read-based SV detection [9]. We benchmarked the resulting call sets against the Genome in a Bottle (GIAB) gold standard [21] using Truvari (v4.0.0) [20] with the following parameters: *p*=0.5, *P*=0.5, *r*= 500, *O*= 0.01. Our evaluation focused on insertions (INSs) and deletions (DELs) larger than 50 bp within the high-confidence regions defined by GIAB.

**Figure 6:**
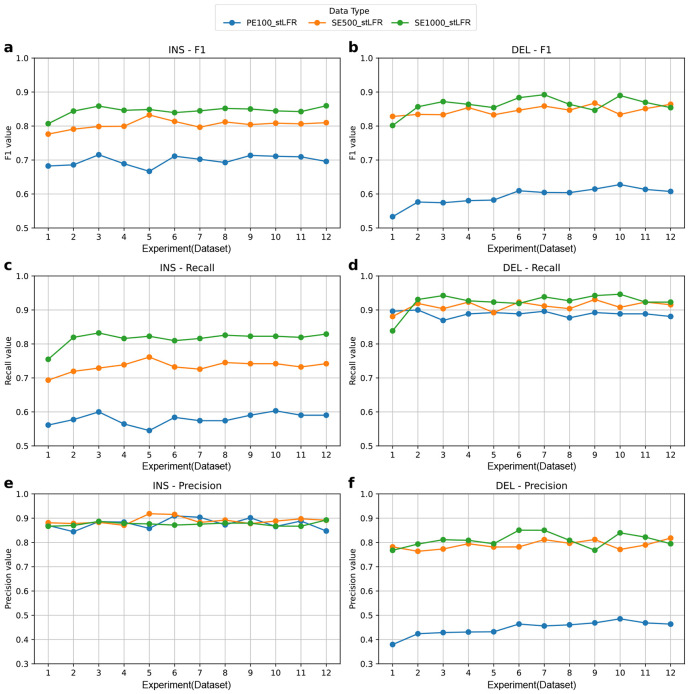
Structural variant detection performance across different single libraries. Aquila_stLFR v1.1 was applied to generate the SV callsets. Truvari v4.0.0 was used to benchmark the result against GIAB gold standard callset (parameters: *p*=0.5, *P*=0.5, *r*=500, *O*=0.01). Libraries are color-coded as follows: blue for PE100_stLFR, orange for SE500_stLFR, and green for SE1000_stLFR. a,c,e show the F1 score, recall, and precision for large insertion (INS) calls **b,d,f** show the F1 score, recall, and precision for large deletion (DEL) calls.

For large INSs, the performance of the three single-library types varies significantly across the evaluated metrics, as shown in Figure 6a, c, and e. SE1000_stLFR demonstrates the highest F1 score, ranging from 0.81 to 0.86 with an average of 0.84 across all 12 datasets, indicating a superior balance between precision and recall. SE500_stLFR follows closely with F1 scores ranging from 0.78 to 0.83 (average 0.80), while PE100_stLFR exhibits the lowest performance, with F1 scores between 0.67 to 0.72 (average 0.70). For recall, SE1000_stLFR again outperforms the others, achieving values between 0.75 and 0.83 (average 0.82), reflecting its ability to detect true insertions. SE500_stLFR shows moderate recall, ranging from 0.69 to 0.76 (average 0.73), whereas PE100_stLFR performs the worst, with recall values between 0.55 and 0.60 (average 0.58), highlighting its limitations in sensitivity. In contrast, precision is highest in SE500_stLFR, ranging from 0.87 to 0.92 with an average of 0.89, reflecting its strong accuracy in identifying true positives. PE100_stLFR follows closely, with a precision range of 0.84 to 0.91 (average 0.88), while SE1000_stLFR, despite its overall superior performance, shows a slightly narrower range of 0.87 to 0.89 (average 0.88). These results suggest that longer read lengths, as in SE1000_stLFR, enhance recall but incur a modest trade-off in precision, whereas shorter reads, such as PE100_stLFR, prioritize precision but struggle with recall.

For large DELs, SE1000_stLFR consistently outperforms other libraries across most metrics (Figure 6b, d, and f). SE1000_stLFR achieves F1 scores ranging from 0.80 to 0.89, with an average of 0.86, indicating strong overall performance. SE500_stLFR is closely competitive, with F1 scores between 0.83 and 0.87 (average 0.85), while PE100_stLFR lags behind, ranging from 0.53 to 0.63 (average 0.59), reflecting reduced balance between precision and recall. In terms of recall, all three datasets perform relatively well: SE1000_stLFR ranges from 0.84 to 0.95 (average 0.92), SE500_stLFR from 0.88 to 0.93 (average 0.91), and PE100_stLFR from 0.87 to 0.90 (average 0.89). These results collectively highlight the robustness of DEL detection across datasets, with SE1000_stLFR showing the most consistent and accurate performance. Precision, however, remains a key differentiator. SE1000_stLFR again leads, with precision values ranging from 0.77 to 0.85 (average 0.81), followed closely by SE500_stLFR, which ranges from 0.76 to 0.82 (average 0.79). In contrast, PE100_stLFR shows substantially lower precision, with values between 0.38 and 0.49 (average 0.45), highlighting a higher rate of false positives in deletion calls. These results emphasize that SE1000_stLFR, benefiting from longer reads, consistently offers the best trade-off between precision and recall. Conversely, the shorter reads of PE100_stLFR appear to hinder accurate deletion detection, particularly in maintaining precision - likely due to the challenges of resolving complex genomic regions with limited sequence context.

Overall, INS calls favor high precision but lower recall, while DEL calls show higher recall at the cost of precision, emphasizing distinct trade-offs between the two SV types. Longer reads are more effective in balancing these metrics, particularly for deletion detection. In contrast, shorter reads such as PE100_stLFR struggle, exhibiting reduced recall for INSs and diminished precision for DELs.

### Simulated and real data comparison

In this session, we compared the sequencing configuration and variant calling performance between our simulated data and real data.

We first compared SNP, small INDEL and SV calling performance between simulated and real PE100_stLFR data. In our simulation experiment, we simulated PE100 stLFR data across 12 different simulation configurations (Table ??). In our simulation pipeline, the determining parameters are $C_{F}$ (coverage of long fragment), $C_{R}$ (coverage of short reads on each long fragment), $\mu_{FL}$ (average fragment length), and sequencing error rate. Specifically, we simulated 12 different libraries for PE100_stLFR, among which $C_{F}$ ranges from 47-350, $C_{R}$ ranges from 0.1-0.75, $\mu_{FL}$ ranges from 50-100kb. The error rate is set to 1% for all 12 libraries. For the real PE100_stLFR data, $C_{F}$ is 931, $C_{R}$ is 0.11, $\mu_{FL}$ is 13.9kb, and the error rate is 1%. For SNP and small INDEL calling (Figure ??), our simulated data on average showed comparable result to real data. In the high-confidence region, the difference between average simulation data F1 and real data F1 is only 0.01 for both SNP and small INDEL calling. In terms of SV calling (Figure ??), the simulated and real PE100_stLFR showed an overall similar trend: for insertion, the precision is high but the recall is relatively lower, while for deletion, the recall is high but the precision is generally lower. One major goal of our simulation is to show how much performance gain stLFR reads can bring when the configuration is in favor of SV calling, so we make the fragment length in the simulated data longer than the most typical length in real data. As a result, the simulated PE100_stLFR dataset achieved a higher average F1 score than the real PE100_stLFR data, consistent with the expectation that longer fragments enhance SV-calling performance.

We further evaluated SNP and small INDEL calling performance using simulated PE100_NGS reads and real Illumina sequencing data. Simulated datasets were generated across 12 configurations (Table ??) at 70× coverage. The two data types exhibited comparable accuracy in both high-confidence and difficult genomic regions (Figure ??), with the mean F1 score of simulated datasets deviating by less than 1% from the corresponding values obtained from real Illumina data across both highconf and alldiff regions. Consistent trends were observed between the real and simulated datasets across individual difficult regions: both exhibited reduced SNP and small indel calling accuracy in lowmap, segdups, and MHC regions, while showing relatively higher accuracy in tandemRep regions. This concordance suggests that our simulation framework effectively reproduces the characteristics of real sequencing data.

## Discussion

In this study, we evaluated the capabilities and limitations of barcoded single-end reads of 500 bp (SE500_stLFR) and 1000 bp (SE1000_stLFR), a conceptual extension of the traditional paired-end linked-read technology (PE100_stLFR), in detecting both small variants (SNPs and INDELs) and structural variants (SVs). Through a series of realistic simulations generated using our novel pipeline, stLFR-sim, we demonstrated that PE100_stLFR consistently outperforms other libraries in SNP and INDEL detection across most genomic regions. This result was somewhat unexpected, as we initially hypothesized that longer single-end reads would perform equally well or better in many regions due to their extended range of sequence information. A key reason for PE100_stLFR’s superior performance is the use of a barcode-aware aligner, whereas no such aligner currently exists for single-end reads. Barcode-aware aligners can resolve ambiguous mappings, particularly in repetitive regions, by leveraging barcode co-localization to rescue reads and enhance mapping confidence. This leads to improved mapping quality, which directly contributes to more accurate variant calling.

However, both SE500_stLFR and SE1000_stLFR offer notable improvements in low-mappability (lowmap) regions, likely due to their increased read lengths. Specifically, the recall rates of SNPs and INDELs in lowmap regions increase greatly with the extension of the read length, suggesting that longer single-end reads can more effectively bridge ambiguous sequences that typically hinder accurate short-read alignment and variant calling.

Given the single linked-read library evaluation results, a natural next step was to investigate whether the complementary strengths of paired-end (PE) and single-end (SE) libraries could be effectively combined. Our hybrid library SNP and INDEL detection evaluations indicated that combining short paired-end reads with longer barcoded single-end reads helps alleviate the limitations of each approach in certain genomic regions. However, the intermediate performance observed in some regions still suggests a persistent trade-off between these benefits, underscoring the need for more refined hybridization strategies or alignment-aware post-processing methods tailored to regional genomic features.

In terms of structural variant detection, our analysis underscores the significant impact of read length on SV calling, clearly demonstrating that longer single-end linked-reads substantially enhance SV detection accuracy compared to shorter paired-end linked-reads. Specifically, SE1000_stLFR consistently provided the optimal balance between precision and recall, enabling robust identification of insertions and deletions greater than 50 bp. SE500_stLFR demonstrated intermediate performance, falling between SE1000_stLFR and PE100_stLFR. While PE100_stLFR reads yielded high precision in insertion detection and high recall in deletion detection, their limited length restricted the recall in insertion detection and precision in deletion detection, resulting overall lower accuracy. These findings emphasize that increased read length in linked-read sequencing markedly improves the resolution of structural complexities, addressing limitations inherent to conventional short-read sequencing methods. Therefore, employing longer single-end reads, or strategically integrating them in hybrid approaches, represents a promising direction for improving comprehensive genomic analyses and structural variant detection in future designs of linked-read sequencing libraries and studies.

## Conclusion

In this study, we systematically investigated the impact of linked-read sequencing designs on the detection of small variants and structural variants in complex genomic regions. Our results reveal clear and consistent trade-offs among different stLFR configurations. PE100_stLFR provides the most reliable performance for SNP and small INDEL calling, whereas long single-end barcoded reads, particularly SE1000, substantially improve structural variant detection by better balancing precision and recall. Hybrid designs further illustrate that combining complementary read types can mitigate individual limitations, offering practical guidance for optimizing linked-read sequencing strategies. Together, this work provides a quantitative framework for evaluating linked-read sequencing strategies and highlights the potential of long single-end barcoded reads as a powerful extension of existing stLFR technology.

## Supplementary Material

This is a list of supplementary files associated with this preprint. Click to download.

• stLFRsimulationpaper20250920revison4.pdf
